# Detection of microbial biofilms inside the lumen of ureteral stents: two case reports

**DOI:** 10.1186/s13256-023-03849-6

**Published:** 2023-04-18

**Authors:** Carolina C. Barajas-García, Alma L. Guerrero-Barrera, José M. Arreola-Guerra, Francisco J. Avelar-González, Flor Y. Ramírez-Castillo

**Affiliations:** 1 Laboratorio de Biología Celular y Tisular, Departamento de Morfología, Universidad Autónoma de Aguascalientes, Aguascalientes, Mexico; 2 Departamento de Nefrología, Hospital Centenario Miguel Hidalgo, Aguascalientes, Mexico; 3 Laboratorio de Ciencias Ambientales, Universidad Autónoma de Aguascalientes, Aguascalientes, Mexico

**Keywords:** Renal transplant, Double J stent, Biofilm, Scanning electron microscopy (SEM), Case report

## Abstract

**Background:**

We report large biofilm structures that covered almost the entirety of the lumen and surface of double-J stents in two postrenal transplant patients, with no development of urinary tract infection. Biofilm bacteria of one patient were integrated by coccus in a net structure, whereas overlapping cells of bacilli were present in the other patient. To the best of our knowledge, this is the first time that high-quality images of the architecture of noncrystalline biofilms have been found inside double-J stents from long-term stenting in renal transplant recipients.

**Case presentation:**

Two renal transplant recipients, a 34-year-old male and a 39-year-old female of Mexican-Mestizo origin, who underwent a first renal transplant and lost it due to allograft failure, had a second transplant. Two months after the surgical procedure, double-J stents were removed and analyzed using scanning electron microscopy (SEM). None of the patients had an antecedent of UTI, and none developed UTI after urinary device removal. There were no reports of injuries, encrustation, or discomfort caused by these devices.

**Conclusion:**

The bacterial biofilm inside the J stent from long-term stenting in renal transplant recipients was mainly concentrated on unique bacteria. Biofilm structures from the outside and inside of stents do not have crystalline phases. Internal biofilms may represent a high number of bacteria in the double-J stent, in the absence of crystals.

## Background

The most successful treatment for end-stage renal disease is renal transplantation [1]. Nonetheless, urological complications may appear during the first 3 months after the surgical procedure, such as urinary leakage, ureteral stenosis, and obstruction, which can be reduced by placing ureteric stents [2, 3].

The use of stents is often related to the colonization and encrustation of these devices, which in turn may lead to serious consequences, such as catheter-associated urinary tract infections (CAUTI) [4]. These phenomena are associated with biofilm formation. Biofilms are highly organized bacterial structures embedded in a self-produced extracellular matrix containing polysaccharides, proteins, lipids, and DNA, among other bacterial macromolecules [5, 6]. Additionally, the presence of urease-producing bacteria that lead to the formation of calcium and magnesium crystals (calcium oxalate, apatite, and struvite) on the intraluminal and external surfaces of stents causes encrustation and blockage of the device, which is advantageous for different types of microorganisms that utilize it as a substrate for adherence and proliferation [7–9]. In addition, most cases of ureteral stent obstruction are related to crystalline biofilms present outside and/or inside the stents [10–13]. Moreover, the presence of chronic medical illness and use of steroids may worsen the prognosis of this uncommon condition [14, 15].

There are several studies about biofilms found in stents from renal transplant recipients and nontransplant patients, primarily focused on species diversity and antimicrobial coatings [16–23]. However, data on the morphological characteristics of bacterial biofilms using high-quality images from the lumen of stents in renal transplant recipients are lacking. Additionally, the images obtained in this report provide new insights into biofilm architecture and extension, since the stents analyzed remained *in situ* for a longer period than usual (2 months).

## Case presentation

**Fig. 1:**
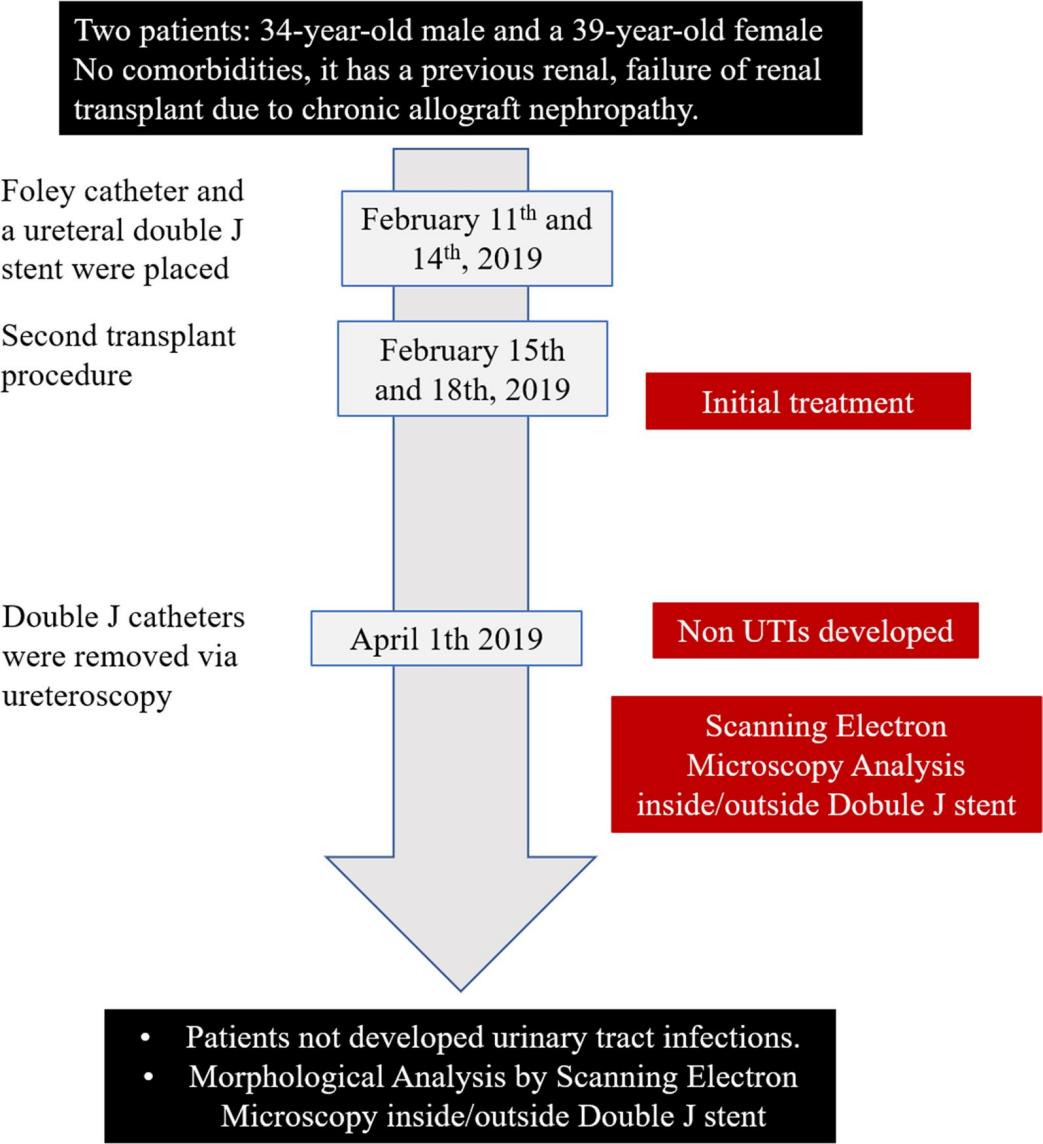
Timeline of interventions and outcomes

Two patients, a male and a female of Mexican-Mestizo origin, who had undergone prior renal transplantation, were admitted at Centenario Hospital Miguel Hidalgo in Aguascalientes, Mexico, for a second renal transplant procedure. Both patients, a 34-year-old male and a 39-year-old female, did not present with comorbidities and suffered graft failure after their first renal transplant due to chronic allograft nephropathy. The second transplantation procedure was performed on 11 and 14 February 2019, respectively. A Foley catheter and a ureteral double-J stent were placed in each patient before transplantation. In both cases, thymoglobulin (3.5 mg/kg, total dose) was administered to induce immunosuppression. Both patients received methylprednisolone (500 mg) before transplantation. On the day after the transplant, the patients received 200 mg, and the dose was gradually reduced until day 5 (25 mg). The same dose was maintained for 1 month and gradually reduced to 5 mg (the final dose, without discontinuation). To maintain immunosuppression, both patients received tacrolimus, mycophenolate mofetil (MMF), and prednisone. Transplant recipients were classified as having a high immunological risk due to second transplant. Approximately 4 days after the transplant, the Foley catheter was removed, and non-urinary tract infection was present. Two months later, the double-J catheter was removed. None of the patients developed UTI or any other kind of infection after transplantation or after urinary device removal, and there were no reports of injuries, encrustation, or discomfort due to the devices. The ureteral stents of both patients were removed on 1 April 2019, via ureteroscopy and placed inside sterile plastic recipients (Fig. 1). The samples were refrigerated (4 °C) until processing (less than a week).

**Fig. 2:**
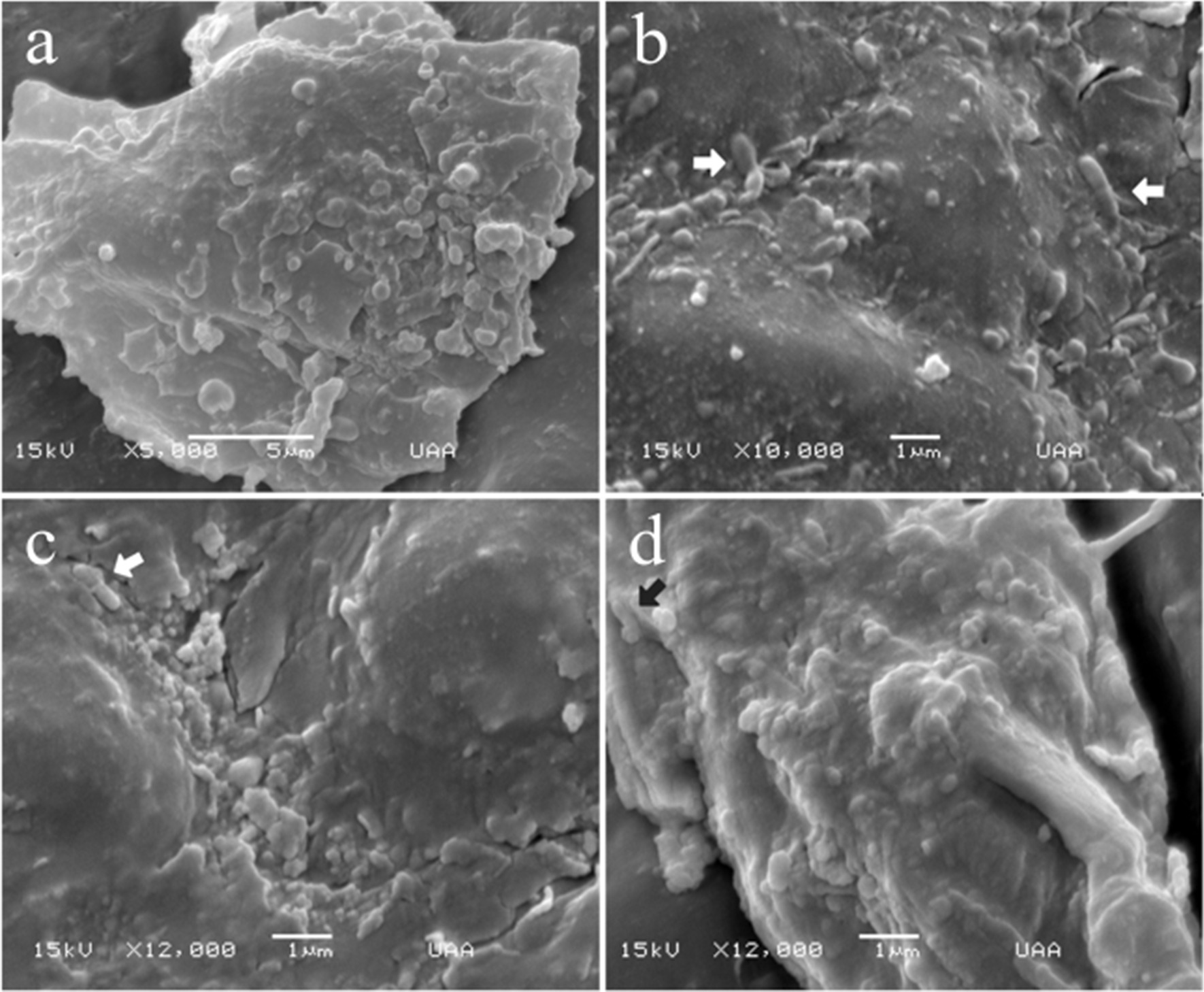
Scanning electron microscopy (SEM) of the biofilm from the external surface of the J stent from male patient. **a** External surface of the J stent, 80×. **b**–**d** Biofilm formation on external surface of J stent. The bacterial cells are shown embedded on extracellular matrix as flat-layered structures. **b** 5000×, **c** 5000×, and **d** 10,000× magnification. No crystalline material was deposited in the bacterial biofilm. Arrows show the bacilli bacteria

**Fig. 3:**
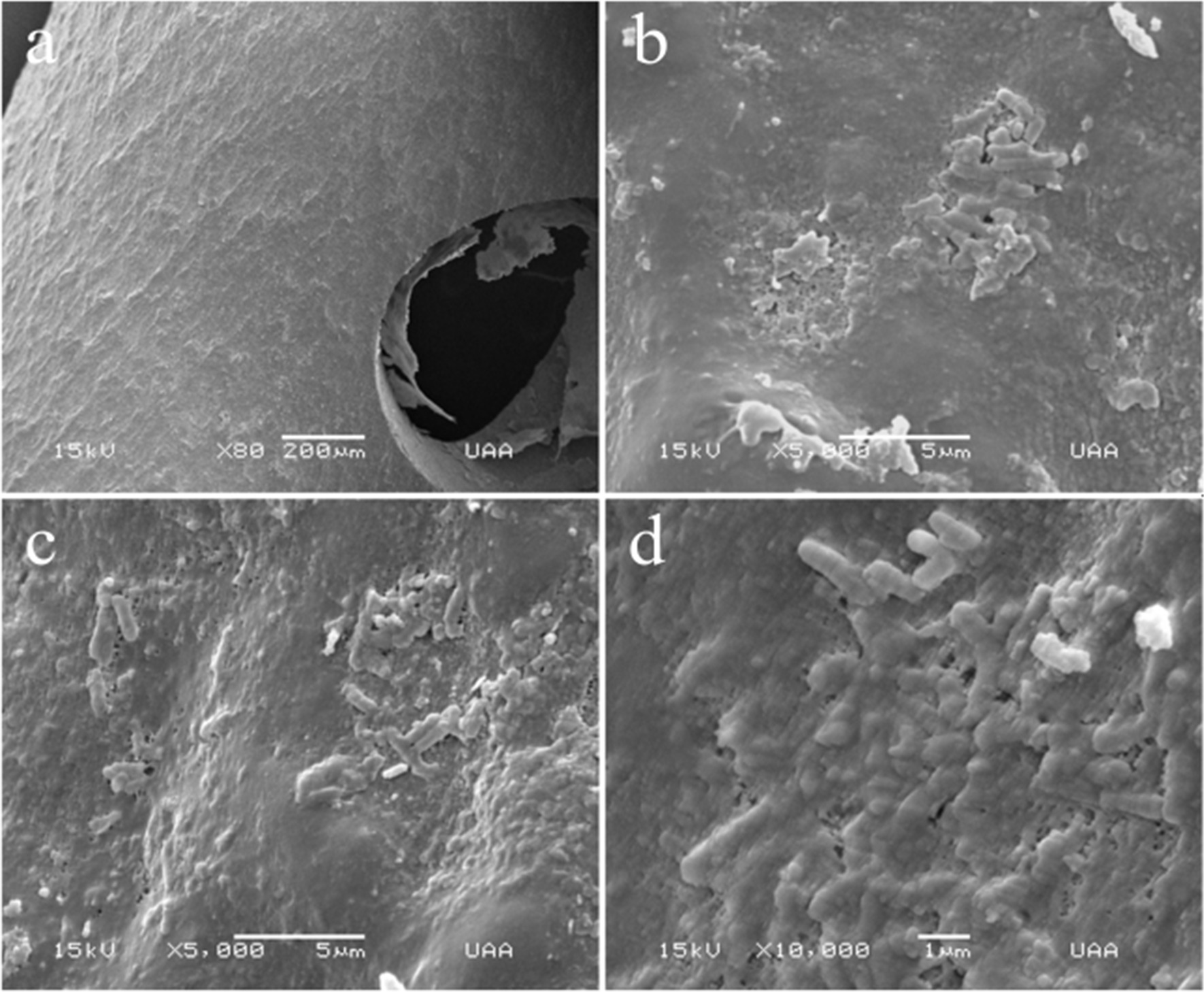
Scanning electron microscopy (SEM) of the biofilm from the external surface of the J stent from female patient. **a**–**d** External surface of the J stent. Heap-shaped, patchy bacterial colonies are shown. Most of the bacteria were embedded on the extracellular matrix. **a** 5000×, **b** 10,000×, **c** 12,000×, and **d** 12,000× magnification, respectively. Arrows show rod-shaped bacteria. No crystalline material was deposited in the bacterial biofilm

Before processing the stents for scanning electron microscopy (SEM), we noticed that both had brown stains randomly distributed on the external surface, and no crystals were found on the external or internal surfaces. SEM of the external surfaces of both stents revealed the presence of biofilms. Patchy bacterial colonies were observed on the external surface of the stent, and heap-shaped bacterial colonies were commonly observed (Figs. 2, 3). Moreover, the external surface of the double-J stent from the male patient displayed some colonies focused on cocci, and some on bacilli (Fig. 2a–d). In contrast, the external surface of the J stent from the female patient (Fig. 3a) displayed mostly *Bacillus* spp. (Fig. 3b–d).

**Fig. 4:**
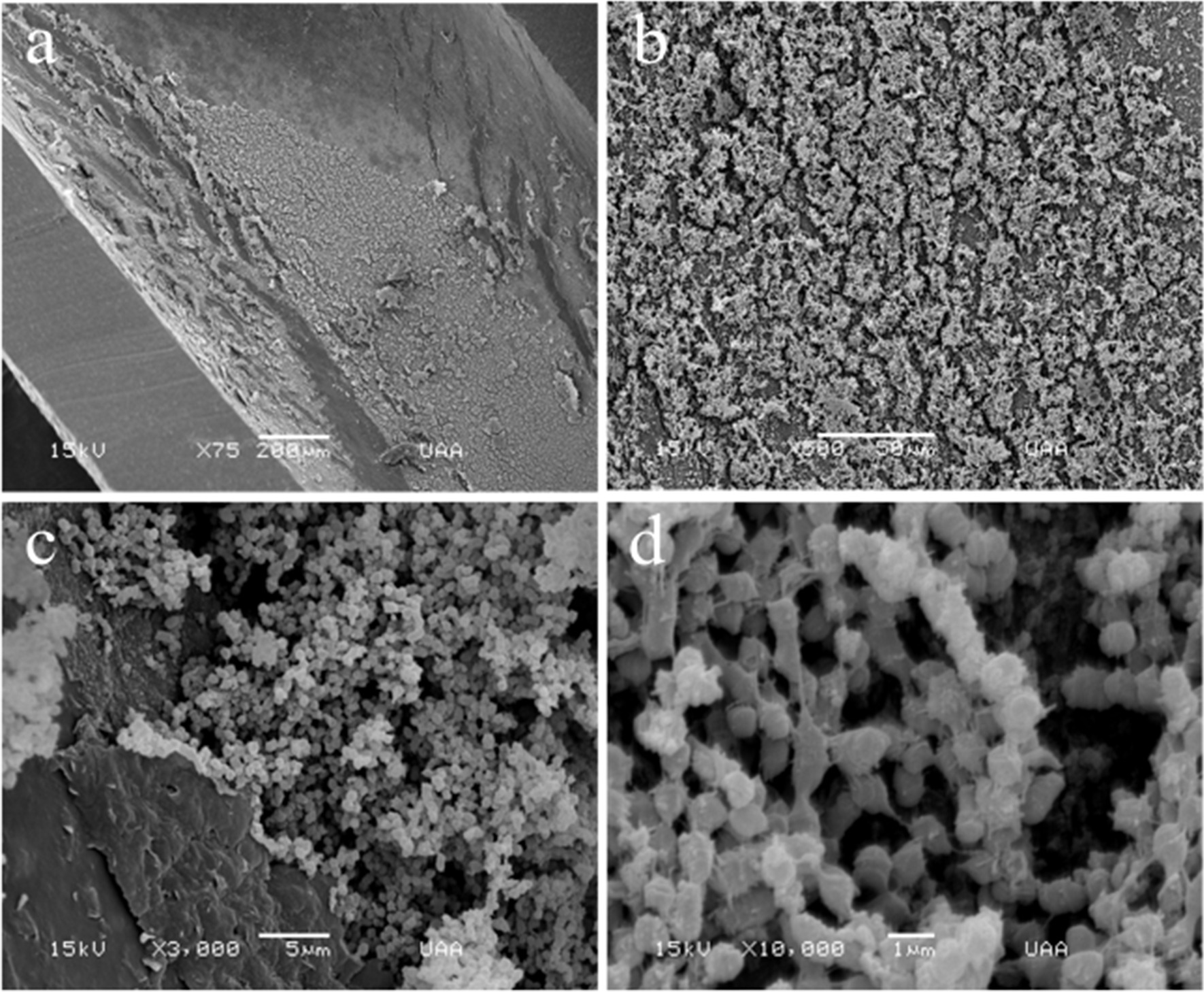
Scanning electron microscopy (SEM) lumen of the J stent from male patient, colonized by coccus bacteria. **a** Luminal surface of a double-J stent cut longitudinally covered by the biofilm. **b** 500× magnification. **c** Aggregation of cells, 3000×. **d** Biofilm architecture, constituted by a net of bacterial cells connected by polysaccharides. No crystalline material was deposited in the bacterial biofilm

**Fig. 5:**
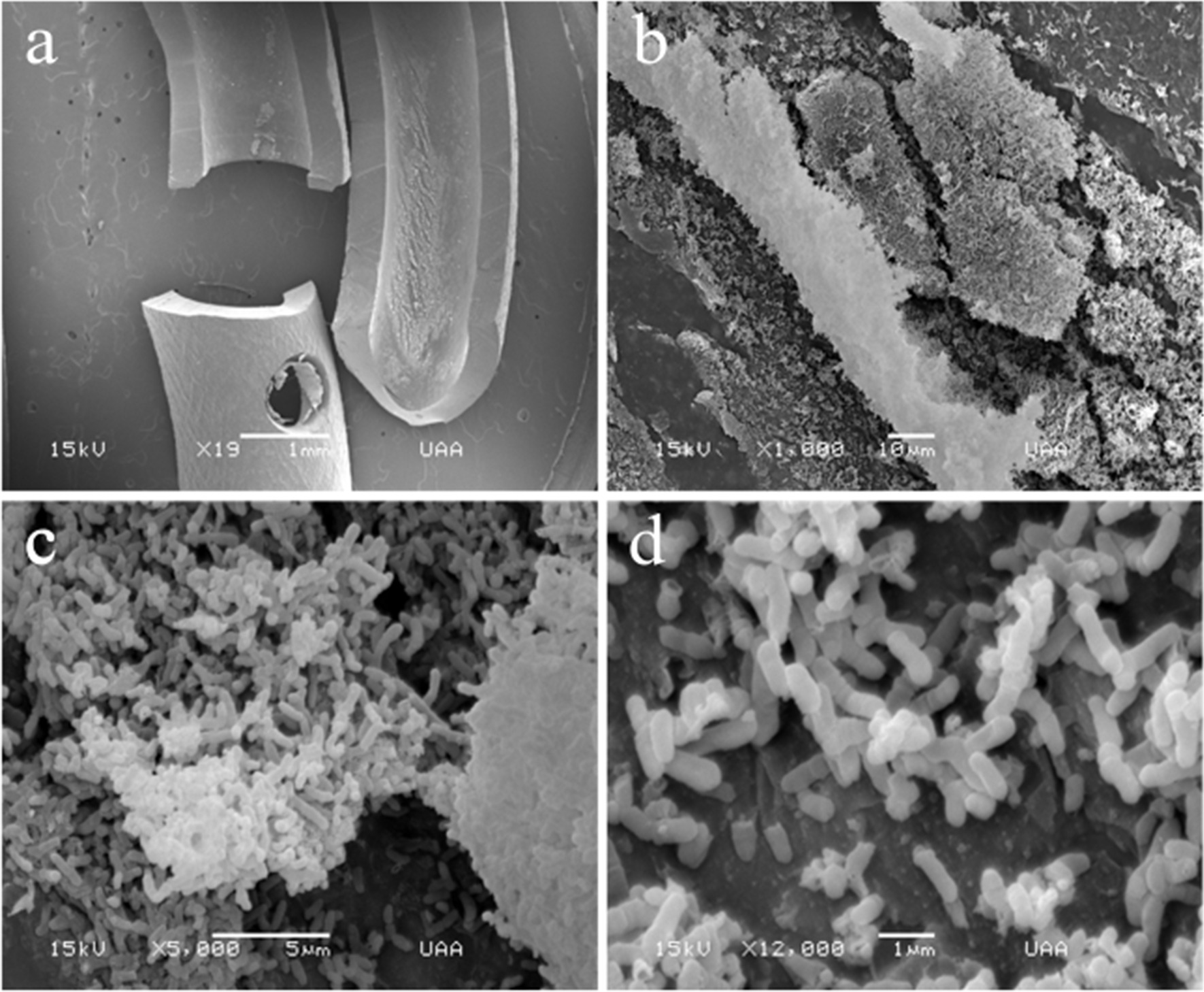
Scanning electron microscopy (SEM) from the lumen of the stent from the female patient, colonized by bacillus bacteria. **a** Luminal surface of a double-J stent cut longitudinally covered by the biofilm. **b** Biofilm architecture, constituted by multi-layered plaques of bacterial cells, 1000× magnification. **c** Aggregation of bacterial cells, 3000×. **d** Rod-shaped cells, 10,000×. No crystalline material was deposited in the bacterial biofilm

Scanning of the lumen of the stents (Figs. 4, 5) was achieved through longitudinal cuts (Figs. 4a, 5a). Light-toned structures that completely covered the internal surfaces of both stents are shown (Fig. 5b). Under further magnification, a large number of biofilm structures were observed. Although both biofilms were multilayered, their internal architectures were different (Figs. 4 and 5). Specifically, the biofilms in the stent lumen of the male patient did not have a determined shape, since they looked like scattered structures (Fig. 4b, c). However, with a magnification of 10,000×, they resembled nets linked with filaments of extracellular polymeric substance matrix (EPS matrix) and were composed of coccus-shaped bacteria with a size range of 0.5–0.8 μm, presumably *Enterococcus faecalis* (*E. faecalis*) (Fig. 4d). We observed fimbriae-like or curli-like structures and that promoted interactions between all the bacteria present in the biofilms (Fig. 4c, d). Additionally, these structures appeared to be more abundant in the coccus biofilm (Fig. 4b–d) than in the bacillus biofilm (Fig. 5b–d). On the other hand, rod-shaped bacteria with a size range of 0.6–1.0 μm, resembling *Escherichia coli* (*E. coli*) (Fig. 5b–d), were found constituting the biofilms on the lumen from the female patient; these structures were formed by overlapped bacterial cells (Fig. 5d). Interestingly, on the external surface of the stent, we observed 3D structures with most bacteria embedded in the extracellular matrix (Figs. 2, 3). In contrast, the lumen of the J stent exhibited a connection between bacteria through fimbriae-like or curli-like structures, and a thicker matrix attached to the bacteria (Figs. 4, 5). Unexpectedly, non-crystalline biofilms were found on the surface (Figs. 2, 3) or in the lumen of the two double-J stents (Figs. 4, 5).

## Discussion

The data presented here are limited by the restriction of clinical practice since methods for the management of biofilms are not fully applied in the clinical environment. We were unable to perform a confirmatory test using 16S rRNA or confocal staining analysis. Despite these limitations, this study provides novel insights into the microbial biofilm in the lumen of stents after long-term stenting. Further characterization of the lumen of the double-J stent of biofilm structures in a higher number of patients is required to determine whether there is an association between biofilm formation inside the lumen and non-crystal development through long-term stenting, as well as whether it is related to only one-species biofilm colonization.

Biofilm formation is enhanced by the presence of catheters or stents, because they function as substrates for planktonic bacteria to adhere to and thus resulting in microcolony growth. These colonies become irreversibly attached with the secretion and accumulation of fibrinogen, caused by the devices, and eventually grow and produce an extracellular matrix composed of polymeric substances to protect themselves [4, 24, 25]. It has been reported that biofilm formation affects up to 50% of the patients with indwelling urinary devices placed for at least 4–7 days, while have been discovered to cover 100% of stents placed for more than 28 days [4, 5]. Major complications related to the use of the double-J stent include encrustation, vesicoureteral reflux, urinary infection, migration, stent fracture, ureteral arterial fistula, and necrosis [26]. Hematuria and extreme pain are known complications of stent encrustation. Previous studies [27, 28] have shown that long-term stent use is associated with infection and precipitation of salts from urine, which can lead to the build-up of crystalline deposits on the stent surface, making stent removal difficult and painful. These studies found ureteral stents covered by biofilm on the 7th and 31st days of retention, with various numbers of inflammatory attachments and encrustation in the outer and inner surfaces of the double-J stents. In addition, previous studies have found a significant association between the duration of double-J stent use, postrenal transplant, and stent colonization [29–31]. It is important to state that even when intraluminal biofilms are not in direct contact with patients’ tissues, they are still reservoirs of bacteria that may be pathogenic and could cause CAUTI [32]. Different studies have reported different frequencies of UTIs among kidney transplant recipients. Ranganathan *et al.* [30] and Abu *et al.* [29] found that the incidence of UTI in patients with stents was significantly higher than that in patients without stents. In contrast, other studies [31–33] found that the incidence of UTI in both groups was similar, or that there was no significant difference in the occurrence of UTI between the two groups. However, recent evidence suggests that the prevalence of asymptomatic bacteriuria is low among kidney transplant recipients beyond the 2nd month posttransplantation [34].

In this case study, double-J stents for two renal transplant recipients were placed for 2 months, a considerable period for biofilm maturation and bacterial dispersal. Despite the risks associated with carrying an indwelling device, the patients in these cases were asymptomatic for UTIs, and neither of them had a history of infection. This is in agreement with recent findings in transplant recipients, during the 2 months after the surgical procedure was performed, indicating that the presence of large numbers of bacteria in urine when the patients are asymptomatic may have no clinical relevance in terms of the development of subsequent UTI [34, 35]. Moreover, asymptomatic bacteriuria is frequently observed in kidney transplant recipients, with an incidence of approximately 40% [29, 34].

There is a paucity of data on the morphological characteristics of bacterial biofilms from the lumen of the stents of renal transplant recipients, and only a handful of reports have displayed high-quality images of the lumen and compared the lumen with the external surface of the J stent. When we compared the structure of the lumen with the external surface of the stent, we noticed that most of the biofilm on the external surface had a 3D structure, water channels, and patchy bacteria embedded in a high amount of extracellular matrix as was found by other authors [4, 12], with mono- and di-species bacteria. In contrast, the lumen of the J-stent revealed bacterial biofilms of only one species that interacted through fimbriae-like/curli-like structures. Low nutrient levels may trigger extracellular matrix production on the external surface compared with the lumen of the J stent, where urine flow is continuous. This is in accordance with Zhang *et al.* [36], who showed that nutrient depletion in *Bacillus subtilis* biofilms triggers matrix production.

Coccus shapes were visualized in male patients, and bacillus shapes were observed in female patients on both sides of the stent. *E. coli* is the most frequently isolated organism on urinalysis during UTIs, and biofilms related to catheters and stents. Other bacterial species commonly found are *Klebsiella pneumoniae*, *Pseudomonas aeruginosa*, *Staphylococcus aureus*, and *E. faecalis* among which, however, the frequency of isolation depends on the geographical zone, since differences have been found in multiple studies [24, 37–40]. In the present study, we found biofilms composed of only one species of bacteria inside the lumen of the double-J stent. On the other hand, bacterial species outside the double-J stents seemed to belong to two species: coccus and bacillus. It is also common to find multispecies biofilms, given that stents remain *in situ* for a long period [8, 41]. The aseptic conditions in which the double-J catheters are placed could be the reason for mono-species biofilms inside the J stent [42].

Interestingly, even in the long-term J stent, large amounts of biofilms were found on the external surface and lumen of the J stent, apparently with noncrystal formation. It has been shown that the formation of mineralized biofilms on stents is mainly due to urease-secreting strains, as they secrete urease, which increases urine pH, resulting in the precipitation of struvite and hydroxyapatite crystals, adhesion factors, transporters, transcription factors, communication factors, enzymes, and a two-component system [43, 44]. In this case, we found bacterial species that were not urease-secreting strains, such as *Escherichia coli* and *Enterococcus faecalis.*

This study provides insights into the morphology of the microbial biofilm inside the lumen of the ureteral stent, as well as the large number of bacteria present in the lumen of the J stent.

## Conclusions

The bacterial biofilm inside the double-J stent, resulting from long-term stenting of renal transplant recipients, mainly concentrated on unique bacteria that interacted with the net-like structures, while the external surface enhanced the production of extracellular matrix with different bacterial species. Internal biofilms may represent a high amount of bacteria in the long-term double-J stent, in the absence of crystals. However, despite the large number of biofilm-forming bacteria, the patients remained asymptomatic. Additionally, this case report contributes to the field of microbial biofilm architecture in stents.

## Data Availability

Data sharing is not applicable to this article, as no datasets were generated or analyzed during the current study.
